# Fatty Acids Analysis of Four *Pistacia* Species by Gas Chromatography Coupled With Mass Spectrometry via Multivariate Chemometrics

**DOI:** 10.1002/cbdv.202501787

**Published:** 2025-09-11

**Authors:** Ahmed Boukelouaa, Maroua Hadji, Hamdi Bendif, Mohamed Boukeloua, Mustafa Abdullah Yilmaz, Hamdi Temel, Fahmi Boufehdja, Stefania Garzoli

**Affiliations:** ^1^ Department of NaturitionSciences Laboratory LGBVIPA, Institute of Applied Sciences and Techniques ISTA AinM'lila, Faculty of Sciences University of Oum EL Bouaghi Oum EL Bouaghi Algeria; ^2^ Department of Natural and Life Sciences, Faculty of Sciences University of M'sila University Pole, Road Bordjbouarreiridj M'sila Algeria; ^3^ Biology Department, College of Science Imam Mohammad Ibn Saud Islamic University (IMSIU) Riyadh Saudi Arabia; ^4^ Laboratoire De Génie des Procédés pour Le Développement Durable Et Les Produits de Santé (LGPDDPS), Ecole Nationale Polytechnique de Constantine; Laboratoire de Biostatistique, Bioinformatique Et Méthodologie Mathématique Appliquées aux Sciences De La Santé (BIOSTIM), Faculté de Médecine Université Salah Boubniderconstantine 3 El Khroub Algeria; ^5^ Department of Analytical Chemistry, Faculty of Pharmacy Dicle University Diyarbakir Turkey; ^6^ Department of Chemistry and Technologies of Drug Sapienza University Rome Italy

**Keywords:** Chemo‐metrics, fatty acids, GC‐MS, hierarchical cluster analysis, *Pistacia*

## Abstract

*Pistacia* species are widely used in traditional medicine, particularly for wound healing. This study investigated the fatty acid composition of fruits from four *Pistacia* species collected from various regions of Algeria. Dried fruits of *Pistacia lentiscus* L. were extracted using hexane in a Soxhlet apparatus. The extracted lipids were subjected to acid hydrolysis and then converted into their corresponding methyl esters by refluxing with methanolic sulfuric acid prior to analysis. These methylated fatty acids were analyzed by gas chromatography coupled with mass spectrometry. The major fatty acids identified were oleic acid (C18:1n9c), palmitic acid (C16:0), linoleic acid (C18:2n6c), palmitoleic acid (C16:1), and stearic acid (C18:0). Multivariate statistical analysis using R software (version 4.3.3), including principal component analysis and hierarchical clustering, was applied to explore patterns among the fatty acid profiles. Oleic acid was dominant in PL3 (51.18%), linoleic acid in PL1 (21.86%), and palmitoleic acid in PL2 (3.26%). These findings support the ethnomedicinal relevance of *Pistacia* species and provide the first detailed chemometric profiling of their fruit fatty acid content.

## Graphical abstract

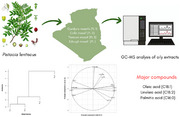

## 1 Introduction

For millennia, the use of medicinal plants has been the primary means of human healing, which was generally adapted to minor illnesses, aiming for symptomatic treatment. There are more than 500,000 plants on Earth, approximately 100,000 of which possess medicinal properties due to their active ingredients that act directly on the body. They are used in both conventional medicine and herbal medicine: they offer benefits that conventional medications often lack [1, 2, 3]. The *Anacardiaceae* family, commonly known as the sumac or cashew family, includes about 860 species and 83 genera around the world [4, 5]. The genus *Pistacia* sp. is spread across North Africa, Southern Europe, West Asia, and North America, and is widely distributed along the Mediterranean basin. Many species, such as *P. lentiscus*, have adapted to Mediterranean semiarid climates and deserts, but also to saline soils. To withstand salt stress, this species evolved as shrubs; however, humidity favours their growth as tree forms [6, 7]. This species is native throughout the Mediterranean region, from Morocco and the Iberian Peninsula in the west, Southern France and Turkey to Iraq and Iran in the east. Several species of the genus *Pistacia* sp. have been used since ancient times as remedies for diseases and consumed as food [8, 9]. Examples of the latter utilities comprise the consumption of pistachio (*P. vera*) nut as a food additive, of *P. terebinthus* fruit as snack food or in making a coffee‐like drink, and of the anthocyanin composition of *P. lentiscus* fruits as food colorants [10, 11].

Due to their edibility, but also use as medicinal plants and natural products, these species have played a very important role in the prevention of diseases and health care for centuries. Several prescribed drugs are obtained nowadays directly or indirectly from plant sources [11, 12]. It was revealed that *Pistacia* species possess a wide range of biological and pharmacological effects. As a result, the extracts derived from different species of the genus *Pistacia* sp. are also currently used in modern medicine [13]. In particular, the fatty oils extracted from *P. lentiscus* berries are used to care for respiratory diseases, diarrhoea, and pharyngitis, as well as in the treatment of wounds and burns [3, 11, 14, 15, 16]. Previous studies also showed that *P. lentiscus* fatty oils have antihyperlipidemic effects [17]. Despite its limited geographic distribution, this plant is nowadays used on a global scale for several therapeutic properties, given its antiulcer, antibacterial, antioxidant, antifungal, and antiproliferative effects. Independent ethnobotanical and ethnopharmacological surveys were conducted in Jordan and Palestine, where the potential of this species in the treatment of stomachaches, heartburn, jaundice, and respiratory problems from lentisk leaves was investigated [18]. On the other side, its fruits were observed to provide edible oil, which is rich in the unsaturated fatty acids oleic and linoleic. Fatty acids have many important biochemical functions, such as energetic, metabolic, and structural activities, but are also known to remediate asthma, liver failure diseases, and rheumatism, due to their antioxidant properties [19, 20, 21].

Although several studies have reported on the biological activities and ethnopharmacological uses of *Pistacia* species, particularly *P. lentiscus*, few have focused on the detailed fatty acid composition of their fruits from different geographical origins. Moreover, there is a lack of chemotaxonomic data that explores the relationship between fatty acid profiles and environmental or geographical variations across Algerian regions. Most available research emphasizes essential oils or polar compounds, overlooking the lipid fraction, which is also of nutritional and pharmacological relevance. Therefore, a comprehensive analysis of the fatty acid content of *Pistacia* fruits, using robust analytical (gas chromatography coupled with mass spectrometry [GC‐MS]) and statistical (PCA and HCA) approaches, is needed to fill this knowledge gap. The aim of the present study was to characterize the fatty acid profiles of fruits from different *Pistacia* species collected across various regions in Algeria using the GC‐MS technique, in order to generate detailed compositional data that may support future investigations. To assess the relationships between the quantified fatty acids, the *Pistacia* samples, and their geographic origins, chemometric methods such as principal component analysis (PCA) and hierarchical cluster analysis (HCA) were applied. The results provide a basis for distinguishing among *Pistacia* species and contribute to the chemotaxonomic understanding of their lipid profiles.

## 2 Results and Discussion

### 2.1 GC‐MS‐Based Profiling of Fatty Acids

The results obtained from the analysis of the extracts PL1, PL2, PL3, and PL4 by GC‐MS are reported in Table 1. The detected compounds correspond to methyl esters of fatty acids (FAMEs), which are commonly used as analytical derivatives in GC‐MS to determine the fatty acid composition of lipid extracts.

**TABLE 1:** Fatty acid composition in *Pistacia* species.

Fatty acids	PL1	PL2	PL3	PL4	Bayes Factor	Conclusion
	%	%	%	%		
**Myristicacid (C14:0)**	ND	0.05 ± 0.01	0.04 ± 0.01	0.08 ± 0.02	1.512	Weak evidence
**Pentadecanoic acid (C15:0)**	ND	0.01 ± 0.005	0.01 ± 0.007	0.03 ± 0.01	1.721	Weak evidence
**Palmitic acid (C16:0)**	20.83 ± 0.63	23.74 ± 0.77	30.02 ± 0.79	11.05 ± 0.55	0.676	Weak evidence
**Palmitoleic acid (C16:1)**	2.91 ± 0.12	3.26 ± 0.21	2.36 ± 0.17	0.38 ± 0.11	1.268	Weak evidence
**Heptadecanoic acid (C17:0)**	0.04 ± 0.01	0.06 ± 0.01	ND	0.055 ± 0.01	0.618	Weak evidence
**Stearic acid (C18:0)**	1.00 ± 0.15	1.29 ± 0.20	1.28 ± 0.17	ND	0.839	Weak evidence
**Oleic acid (C18:1n9c)**	48.91 ± 0.93	50.21 ± 1.24	51.18 ± 1.33	31.49 ± 0.86	0.925	Weak evidence
**Linoleic acid (C18:2n6c)**	21.86 ± 0.37	20.48 ± 0.33	14.52 ± 0.11	18.34 ± 0.29	0.871	Weak evidence
**Linolenic acid (C18:3n6)**	0.30 ± 0.06	ND	ND	0.21 ± 0.02	0.637	Weak evidence
**Arachidic acid (C20:0)**	0.10 ± 0.01	0.15 ± 0.01	ND	0.41 ± 0.01	0.784	Weak evidence
**Cis‐11‐Eicosenoic acid (C20:1)**	0.11 ± 0.01	ND	ND	0.31 ± 0.01	0.751	Weak evidence
**Lignoceric acid (C24:0)**	0.04 ± 0.06	ND	ND	0.90 ± 0.07	0.994	Inconclusive
**∑SFA** ^[^ ^a^ ^]^	22.01 ± 0.55	25.30 ± 0.71	31.35 ± 0.78	12.52 ±	—	—
**∑MUFA** ^[^ ^b^ ^]^	51.93 ± 0.47	53.47 ± 0.94	53.54 ± 0.97	32.18 ±	—	—
**∑PUFA** ^[^ ^c^ ^]^	22.16 ± 0.33	20.48 ± 0.29	14.52 ± 0.12	18.55 ±	—	—
**∑PUFA/∑SFA**	1.007 ± 0.04	0.809 ± 0.02	0.46 ± 0.01	1.48 ±	—	—

ND: not detected.

^[a]^ Saturated fatty acids.

^[b]^ Monounsaturated fatty acids.

^[c]^ Polyunsaturated fatty acids.

For PL1 and PL4, the main fatty acids identified were oleic acid (48.91% and 31.49%, respectively), followed by linoleic acid (21.86% and 18.34%) and palmitic acid (20.83% and 11.05%). In contrast, for PL2 and PL3, the dominant fatty acids were oleic acid (50.21% and 51.18%), palmitic acid (23.74% and 30.02%), and linoleic acid (20.48% and 14.52%). The samples PL1, PL2, and PL3 also contained lower proportions of palmitoleic acid (C16:1) at 2.91%, 3.26%, and 2.36%, respectively, as well as stearic acid (C18:0) at 1.00%, 1.29%, and 1.28%. Other fatty acids were detected in minor amounts (<0.42%), some of which remain unidentified.

At this stage of the study, the results indicate that unsaturated fatty acids (both mono‐ and polyunsaturated) dominate the composition of the extracts, as shown in Table 1. The ratios of total polyunsaturated fatty acids to total fatty acids (∑PUFA/∑FAS) for all PL oily extracts range from 0.46 to 1.48. Notably, PL4 (1.48) and PL1 (1.007) exhibit ∑PUFA/∑FAS ratios greater than or equal to 1, respectively—an indicator of good nutritional quality, as values near or above 1 are considered beneficial according to recognized dietary recommendations [22].

A study on lentisk oil reported a similar profile of major fatty acids. Subsequent research on the nonpolar extract of PL fruits also identified oleic, linoleic, and palmitic acids as the dominant fatty acids. Thus, our findings on the fatty acid profile of PL fruits are consistent with previously published results where the oleic (46.91%–48.34%), palmitic (25.44%–25.75%), and linoleic acids (20.27%–20.59%) were the predominant fatty acids in both hexane and cold‐pressed extracts of *P. lentiscus* fruits [14, 16, 23]. Similarly, lipid accumulation during fruit maturation reported an increase in oleic acid content from 19.49% to 50.72% and a corresponding decrease in linoleic acid from 42.5% to 21.75%, while palmitic acid remained a major component. At full maturity, oleic acid was the most abundant, followed by palmitic and linoleic acids. The study by Charef et al. [23] further supported these trends, showing that black fruits of *P. lentiscus* contained high levels of oleic acid (55.3%–64.9%), along with palmitic (16.3%–19.5%) and linoleic (17.6%–28.4%) acids, highlighting the predominance of unsaturated fatty acids (78.8%–83.5%). These consistent observations across various studies, despite differences in extraction methods or fruit maturity stages, underscore the typical fatty acid profile of *P. lentiscus* oil and confirm its richness in monounsaturated and polyunsaturated fatty acids. In addition, when compared with other Pistacia species and varieties, some notable similarities and variations emerge. For example, reported that oleic acid was the major fatty acid in seven *Pistacia vera* varieties from southeastern Turkey, with values ranging from 53.98% to 68.27%, followed by linoleic acid and palmitic acid [24]. Similarly, Tavakoli and Pazhouhanmehr [25] found high levels of oleic (around 56%–59%) and linoleic acids in the oils of *P. atlantica*, *P. vera*, and *P. khinjuk* growing in Iran. Our study aligns with these results in that oleic and linoleic acids were also the dominant components, supporting the idea that unsaturated fatty acids are predominant across various *Pistacia* species [26] analyzed the regiospecific distribution of fatty acids in *P. vera* oils from seven Iranian varieties and noted a high monounsaturated‐to‐polyunsaturated fatty acid ratio, a trait that we also observed in *P. lentiscus*, although the specific proportions differ slightly due to species and environmental differences. Furthermore, the work on the lipid profiles of *P. terebinthus, P. atlantica*, and *P. khinjuk* also highlighted oleic acid as the major fatty acid in kernels, ranging from 47.2% to 59.6%, which is comparable to our findings [27]. More recently, a study by Gündüz et al. [28] examined eight unprocessed *P. vera* varieties from Spain and confirmed that oleic acid remained the most abundant, followed by linoleic and palmitic acids. This consistency across studies underscores a shared lipid pattern among *Pistacia* species, though minor quantitative differences are likely influenced by genetic variation, climatic factors, and ripening stages. Our results therefore enrich the current understanding of *Pistacia lentiscus* lipid composition and reinforce its nutritional relevance when compared with other well‐studied members of the genus (Table 2).

**TABLE 2:** Correlation matrix of the fatty acid concentrations in the *Pistacia* species.

	C14:0	C15:0	C16:0	C16:1	C17:0	C18:0	C18:1n9c	C18:2n6c	C18:3n6	C20:0	C20:1
C15 :0	**0.94**										
C16 :0	−0.47	**−0.68**									
C16 :1	**−0.71**	**−0.90**	**0.73**								
C17 :0	0.30	0.30	**−0.70**	−0.12							
C18 :0	**−0.60**	**−0.82**	**0.93**	**0.93**	−0.42						
C18 :1n9c	**−0.70**	**−0.89**	**0.92**	**0.94**	−0.45	**0.99**					
C18 :2n6c	−0.39	−0.33	−0.39	0.33	**0.76**	−0.04	0.01				
C18 :3n6	−0.32	−0.01	**−0.65**	−0.31	0.31	**−0.56**	−0.44	**0.56**			
C20 :0	**0.70**	**0.84**	**−0.95**	**−0.79**	**0.69**	**−0.92**	**−0.95**	0.22	0.40		
C20 :1	0.48	**0.73**	**−0.94**	**−0.88**	0.43	**−0.99**	**−0.96**	0.13	**0.66**	**0.89**	
C24 :0	**0.72**	**0.91**	**−0.88**	**−0.95**	0.40	**−0.98**	**−0.99**	−0.06	0.40	**0.93**	**0.94**
**Values in bold have high correlations with each other, with absolute values higher than 0.5**.											

### 2.2 Principal Component Analysis

Table 3 presents the PCA results based on the quantified fatty acids in various *Pistacia* species collected from different localities.

**TABLE 3:** Contribution of fatty acids to the three principal components.

Fatty acids	Principal component 1	Principal component 2	Principal component 3
C14 :0	5.89	**12.82**	**15.66**
C15 :0	9.32	7.87	2.43
C16 :0	**10.71**	4.50	0.04
C16 :1	9.94	4.39	5.66
C17 :0	3.32	**13.04**	**33.13**
C18 :0	**11.74**	0.03	2.59
C18 :1n9c	**12.04**	0.12	0.32
C18 :2n6c	0.05	**35.97**	6.06
C18 :3n6	2.48	**19.44**	**25.02**
C20 :0	**11.46**	0.43	3.64
C20 :1	**11.14**	0.83	5.02
C24 :0	**11.89**	0.55	0.42
Eigen value	8.24	2.56	1.18
Variance (%)	68.74	21.41	9.83
Cumulative (%)	68.74	90.16	≈100

The PCA revealed that three principal components (PC1, PC2, and PC3) explained the majority of the variation in the dataset, with eigenvalues greater than 1. Specifically, PC1 accounted for 68.75% of the variation, PC2 explained 21.41%, and PC3 contributed 9.84%. These components were strongly influenced by specific fatty acids, which were identified as the main contributors in the dataset, as indicated in Table 3. The bold values highlight the fatty acids with the greatest contributions to each principal component.

**FIGURE 1:**
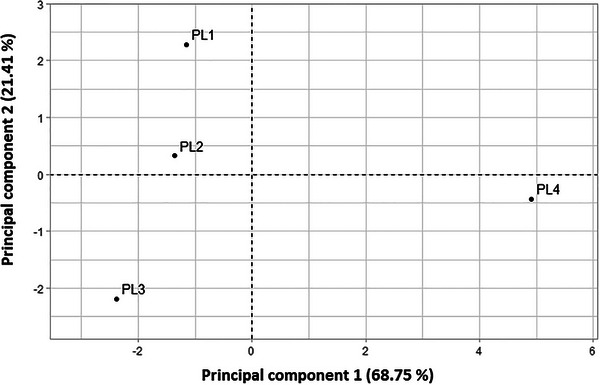
Score plot of PC1 and PC2 for *Pistacia* fruit samples based on fatty acid composition.

PC1 was dominated by Oleic acid (C18:1n9c), Lignoceric acid (C24:0), Stearic acid (C18:0), Arachidic acid (C20:0), Cis‐11‐Eicosenoic acid (C20:1), and Palmitic acid (C16:0). These fatty acids were found in the highest concentrations in PL4 and the lowest concentrations in PL3, as demonstrated in the score values of the first principal component (Table 4). This pattern is supported by the ordination plot (Figure 1), where PL4 is positioned to the right, indicating a higher concentration of these fatty acids compared to PL1, PL2, and PL3, which are located on the left side of the plot.

**TABLE 4:** Principal component scores of fatty acid profiles in *Pistacia* species.

Samples	Principal component 1	Principal component 2	Principal component 3
PL 1	−1.16	**2.28**	−0.97
PL 2	−1.37	0.33	**1.79**
PL 3	−2.38	−2.18	−0.73
PL 4	**4.90**	−0.42	−0.08

‐ Bold values represent the samples with the highest levels of the main fatty acids listed in the preceding table.

‐ Italicized values represent the samples with the lowest levels of those same dominant fatty acids.

PC2 was characterized by Linoleic acid (C18:2n6c), Linolenic acid (C18:3n6), Heptadecanoic acid (C17:0), and Myristic acid (C14:0). These fatty acids were most concentrated in PL1, with the lowest concentrations observed in PL3. The score values for the second principal component (Table 4) also highlight this trend, indicating that PL1 has the highest concentrations of these fatty acids, while PL3 shows the lowest.

Similarly, PC3 revealed a dominance of Heptadecanoic acid (C17:0), Linolenic acid (C18:3n6), and Myristic acid (C14:0), with the highest concentrations found in PL2. This trend is confirmed by the values of score for the third principal component (Table 4), where PL2 presents the highest concentrations of these fatty acids, while PL1 exhibits the lowest.

The score plot (Figure 1) further confirms these observations, with PL4 positioned on the right side, corresponding to higher concentrations of Oleic acid (C18:1n9c), Lignoceric acid (C24:0), Stearic acid (C18:0), Arachidic acid (C20:0), Cis‐11‐Eicosenoic acid (C20:1), and Palmitic acid (C16:0). Conversely, PL1, PL2, and PL3 are located on the left side of the plot, which corresponds to lower concentrations of these fatty acids. This suggests that PL4 has a distinct fatty acid profile compared to the other samples, particularly in terms of the fatty acids explained by PC1.

These variations in fatty acid concentrations can be attributed to several factors, including environmental conditions such as soil composition, plant growth stages, and other ecological factors. Previous studies have also reported similar patterns in fatty acid compositions among different plant parts, supporting the notion that these variations are not only due to genetic differences but also influenced by external factors such as soil mineral content and environmental conditions [14, 17]. Moreover, factors like synergistic and antagonistic interactions between soil components, as well as the uptake and translocation rates of fatty acids, can also play a crucial role in shaping the fatty acid profiles in plants [29, 30].

**FIGURE 2:**
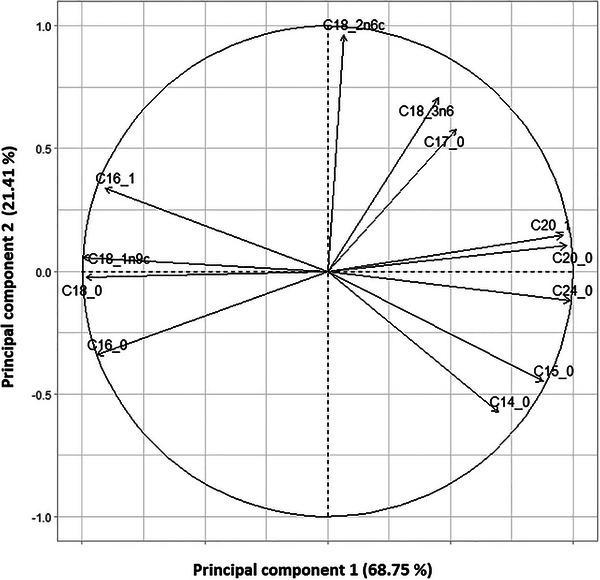
Loading plot of fatty acids on the first two principal components for *Pistacia* species.

The loading plot (Figure 2) illustrates the contribution of each fatty acid to the first two principal components (PC1 and PC2), which together explain 89.89% of the total variance (PC1: 68.75%, PC2: 21.14%). Fatty acids such as C14:0, C16:0, and C24:0 show strong positive loadings on PC1, indicating their significant contribution to the variation captured by this axis. In contrast, polyunsaturated fatty acids like C18:2n6c and C18:3n3 are more correlated with PC2. This distribution highlights the variability in fatty acid composition across *Pistacia* species, suggesting differences in lipid profiles that may relate to species‐specific metabolic or ecological traits.

### 2.3 Cluster Analysis

**FIGURE 3:**
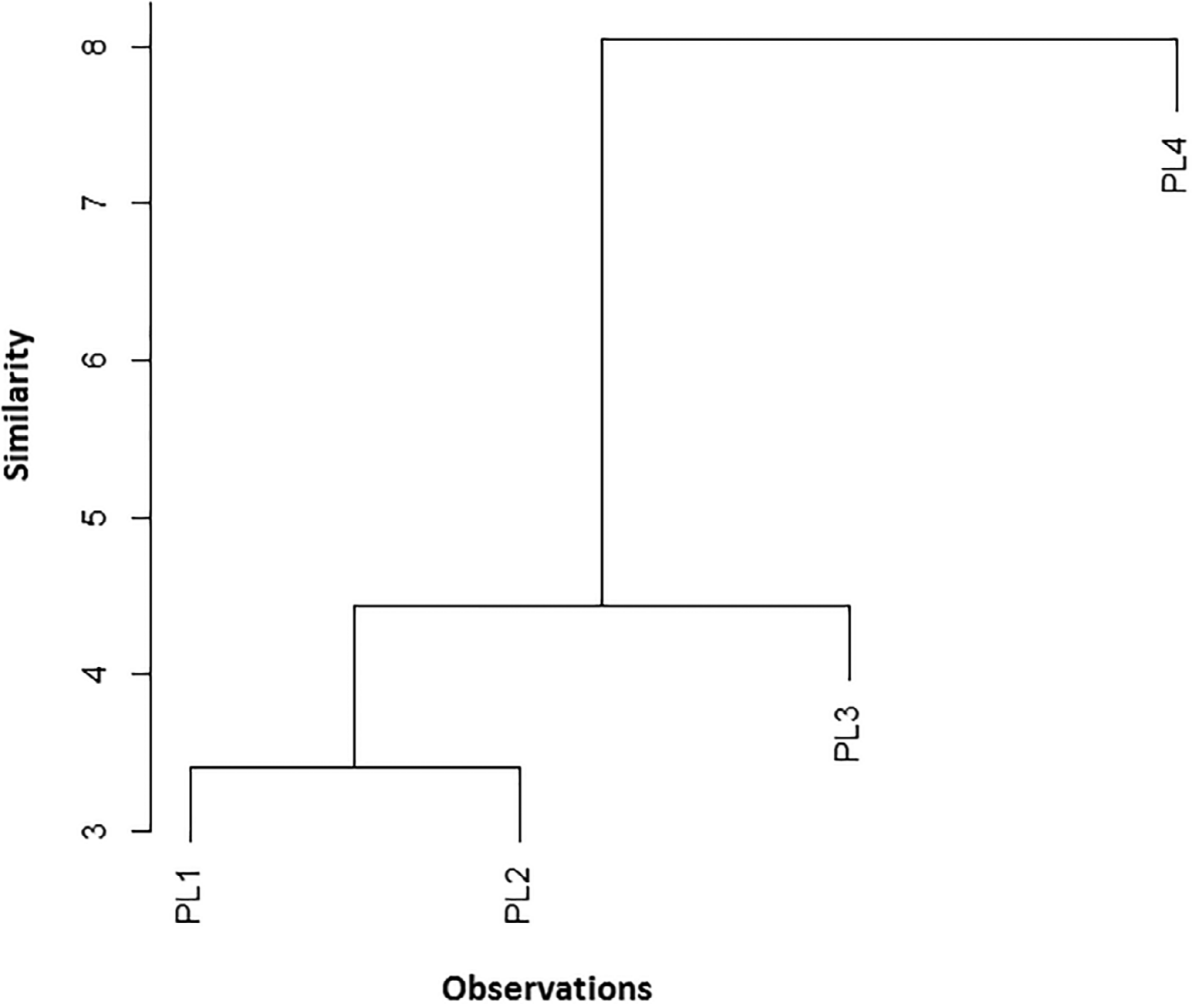
Dendrograms Based on euclidean distance and ward linkage method.

The dendrogram of the HCA is given in Figure 3 and was applied to assess the similarities of samples belonging to various species; each was divided into four fruit parts. HCA was conducted to compare the fatty acid distribution patterns across all the analyzed samples. Afterward, the HCA output was compared to that of the PCA. The data obtained for the 12 studied fatty acids were evaluated using cluster analysis. Figure 3 represents the dendrogram of the HCA applied to the principal component score matrix. The classification and measurement methods were based on squared Euclidean distances and the Ward method, respectively. The HCA analysis revealed three distinct groups: Group 1: PL1 and PL2; Group 2: PL3; Group 3: PL4. This classification aligns with the PCA results, confirming consistent patterns in fatty acid distribution. The grouping of PL1 and PL2 may reflect similar compositional profiles, possibly due to comparable environmental conditions or genetic factors, whereas PL3 and PL4 form separate clusters, indicating distinct lipid profiles.

## 3 Conclusions

It emerges first of all from reading our results that the hexane extracts of PL have interesting compositions in polyunsaturated fatty acids since they have ratios (PUFA/AGS) around 1 (0.46; 0.809; 1.007; 1.48, respectively). It is worth noting that olenic acid (C18: 3, n6) is predominant in the different analyzed samples of PL (51.18/50.21/48.91/31.49% respectively total FA); and Palmitic acid (C16:0) 30.02% total FA for PL3. Followed by Linoleic acid (C18:2n6c) (between 14.52 and 21.86% respectively total FA) for all samples and Palmitic acid (C16:0) (between 11,05 and 20.83% respectively total FA) for the three remaining samples. Other fatty acids are present in lesser amounts (< 0.42%), some of which remain unidentified. The rate of SFAs found for PL4 is three times that measured for PL3. GC‐MS analysis revealed that all examined species are viable for medicinal or nutritional applications due to their fatty acid concentration ratios were consistently in agreement with the recognized dietary recommendations limits for edible plants about the good nutritional value of the oil. The findings revealed intimate correlations among the levels of fatty acid content in different species collected from various localities in Algeria. PCA and HCA techniques, based on the fatty acid concentrations, revealed that there were strong relationships among the investigated species. The species of the genus *Pistacia* sp. were classified into three groups by HCA. Fatty acids with high importance for humans, such as Oleic acid (C18:1n9c), Linoleic acid (C18:2n6c), and Palmitic acid (C16:0), are present in high concentrations. Statistically significant differences were also observed among samples and their geographic location. Since all four investigated species of the genus *Pistachia* sp. used in the current research were collected from different locations, the observed differences in the fatty acid contents equally reflect different environmental conditions, such as soil and climate. The well‐established overall conditioning supply of nutritional compounds, such as polyunsaturated fatty acids, would contribute to more efficiency in the overall process of valorizing the studied extracts. These data emphasize that all the species studied are rich in valuable nutritional components, making them suitable for consumption as part of the recommended daily intake. These results constitute a first indication of the presence of properties of oil composition similarities of the seeds of different PL, under the experimental conditions and limits of this study, which support the medicinal and nutritional properties reported by traditional medicine.

## 4 Experimental

### 4.1 Plant Materials

Fruits of *Pistacia* species were collected in northern Algeria from the thermo‐Mediterranean zone, including the Tell Atlas and forested areas, specifically from four littoral regions: the Djurdjura Massif in Kabylia, the Collo Massif, the Tlemcen Massif, and the Edough Massif west of Annaba. Prof. Houcine Laouer (University of Sétif, Algeria) identified the plant material in 2023. The fruits were washed with distilled water and dried at 70 °C for 48 h [20, 30, 31]. Voucher specimens were collected, identified, and deposited in the Herbarium of Constantine University. The species, codes, and collection sites of the *Pistacia* samples are listed in Table 5.

**TABLE 5:** Details of *Pistacia* Species.

*Pistacia*species	Abbreviations	Collection localities	Latitude (N)	Longitude (E)	Elevation (m)	Herbarium numbers
*Pistacia lentiscus*	PL1	Djurdjura massifs	36.4836°	4.1821°	870 m	PL‐1016AB
*Pistacia lentiscus*	PL2	Collo massif	36.8964°	6.6104°	780 m	PL‐1115AB
*Pistacia lentiscus*	PL3	Tlemcen massif	34.8828°	1.3169°	815 m	PL‐0917AB
*Pistacia lentiscus*	PL4	Edough massif	36.9221°	7.7782°	660 m	PL‐1117AB

### 4.2 Hexane Extraction

The dried fruits of *Pistacia lentiscus* (PL) were ground into a fine, uniform powder using an electric knife mill. Hexane extraction was then performed using a Soxhlet apparatus, operating in three cycles of 8 h each, until the material was fully exhausted. The hexane extracts—oily in appearance—were filtered and concentrated to dryness under reduced pressure using a Heidolph G3 rotary evaporator. This process yielded greenish‐yellow oily residues, which were designated as PL1, PL2, PL3, and PL4. The resulting extracts were stored in amber glass containers at approximately 6°C until further analysis.

### 4.3 Isolation of the Free Fatty Acid Fraction

Isolation of the free fatty acid fraction (AGL) from the hexane extracts (EHPL) was achieved through an acid hydrolysis step followed by esterification, in accordance with previously established protocols [14, 32, 33].

In the procedure, the EHPL extract is acidified to a pH of 1 by the addition of a dilute hydrochloric acid solution. The fatty acids are subsequently extracted by performing three consecutive extractions with 100 mL portions of ethyl ether in a separating funnel. Following filtration and solvent removal under reduced pressure at temperatures below 40°C using a rotary evaporator, a pasty residue is obtained.

### 4.4 Analysis of Fatty Acids by GC‐MS

#### 4.4.1 Preparation of FAMEs

The free fatty acids were esterified by refluxing with 10 mL of a 2% sulfuric acid (H_2_SO_4_) solution in absolute methanol (v/v) for 6 h. Afterward, 20 mL of saturated sodium chloride (NaCl) solution was added to the reaction mixture. The solution was then extracted three times with 15 mL portions of hexane. The combined organic extract was dried using anhydrous sodium sulfate, filtered, and evaporated under reduced pressure and temperature. The resulting residue, consisting of FAMEs, was analyzed by GC‐MS to enable their identification and quantification.

#### 4.4.2 Analysis of FAMEs

Fatty acid analysis was performed using gas chromatography with a flame ionization detector (GC‐FID, Perkin Elmer, Waltham) coupled to a Jeol‐MS mass spectrometer employing electron ionization. The chromatographic separation was achieved on a Restek DB‐5 silica capillary column (30 m × 0.25 mm ID, 0.25 µm film thickness), with nitrogen (N_2_) as the carrier gas at a flow rate of 1 mL/min. The transfer line, injector, and mass spectrometry detector were maintained at temperatures of 220°C and 290°C, respectively. A sample volume of 2.0 µL, dissolved in hexane, was injected. Ionization was carried out at 70 eV. The GC oven temperature was initially set to 60°C for 5 min, then increased by 4°C/min to a final temperature of 240°C, which was maintained for 10 min. The scan time was 0.5 s, with a delay of 0.1 s. Identification of the FAMEs was carried out by comparing their retention times and mass spectra to those of standards using an internal database (LabSolution). The concentration of FAMEs was expressed as a percentage of the total fatty acid content [16, 32].

#### 4.4.3 Data Analysis Methods

The chemometric analyses of the fatty acid content were carried out using two commonly employed multivariate techniques: PCA and HCA. These methods enable classification and clustering based on similarities among samples and relationships among variables. PCA reduces data dimensionality by transforming correlated variables into a smaller number of uncorrelated components, highlighting the main patterns in the dataset. In contrast, HCA groups samples based on their similarity, measured here using Euclidean distances, and presents the results in the form of a dendrogram for easy visualization of clusters. In this study, PCA was applied to differentiate and classify the fatty acid profiles of the plant extracts, allowing identification of the main contributing variables and underlying trends. HCA was also employed to explore the similarities between samples based on their fatty acid composition. Data analysis was performed using the PIROUETTI 2.2 software (Infometrix, Seattle, WA). These approaches are widely used in the analysis of chemical datasets due to their ability to uncover latent structures and patterns [33, 34, 35, 36, 37]. In addition to multivariate analyses, Bayesian statistics were used to assess differences in the relative abundance of individual fatty acids among Pistacia lipid profiles. Bayes Factors (BF_10_) were calculated using a Bayesian one‐way ANOVA framework implemented in the BayesFactor package in R. The analysis compared group means across the four *Pistacia* samples (PL1–PL4) for each fatty acid.

## Author Contributions

**Ahmed Boukelouaa**: conceptualization, investigation, methodology, and writing‐original draft preparation. **Hamdi Bendif**: writing‐original draft preparation, funding acquisition, resources, and supervision. **Mohamed Boukeloua**: data curation, formal analysis; **Maroua Hadji**: writing‐original draft preparation. **Mustafa Abdullah Yilmaz**: validation and data curation. **Maroua Hadji**: methodology, conceptualization, and validation. **Fahmi Boufehdja**: resources and project administration. **Stefania Garzoli**: supervision, writing–review, and editing.

## Conflicts of Interest

The authors declare no conflicts of interest.

## Data Availability

The data that support the findings of this study are available from the corresponding author upon reasonable request.
